# Methane-Derived Carbon as a Driver for Cyanobacterial Growth

**DOI:** 10.3389/fmicb.2022.837198

**Published:** 2022-04-01

**Authors:** Slawek Cerbin, Germán Pérez, Michał Rybak, Łukasz Wejnerowski, Adam Konowalczyk, Nico Helmsing, Suzanne Naus-Wiezer, Marion Meima-Franke, Łukasz Pytlak, Ciska Raaijmakers, Witold Nowak, Paul L. E. Bodelier

**Affiliations:** ^1^Department of Hydrobiology, Faculty of Biology, Adam Mickiewicz University, Poznań, Poland; ^2^Department of Microbial Ecology, Netherlands Institute of Ecology (NIOO-KNAW), Wageningen, Netherlands; ^3^Department of Water Protection, Faculty of Biology, Adam Mickiewicz University, Poznań, Poland; ^4^Department of Aquatic Ecology, Netherlands Institute of Ecology (NIOO-KNAW), Wageningen, Netherlands; ^5^Montanuniversität Leoben, Applied Geosciences and Geophysics, Leoben, Austria; ^6^Department of Terrestrial Ecology, Netherlands Institute of Ecology (NIOO-KNAW), Wageningen, Netherlands; ^7^Molecular Biology Techniques Laboratory, Faculty of Biology, Adam Mickiewicz University, Poznań, Poland

**Keywords:** methane, methane oxidation, isotopes, co-culture, lakes, greenhouse gases, cyanobacteria

## Abstract

Methane, a potent greenhouse gas produced in freshwater ecosystems, can be used by methane-oxidizing bacteria (MOB) and can therefore subsidize the pelagic food web with energy and carbon. Consortia of MOB and photoautotrophs have been described in aquatic ecosystems and MOB can benefit from photoautotrophs which produce oxygen, thereby enhancing CH_4_ oxidation. Methane oxidation can account for accumulation of inorganic carbon (i.e., CO_2_) and the release of exometabolites that may both be important factors influencing the structure of phytoplankton communities. The consortium of MOB and phototroph has been mainly studied for methane-removing biotechnologies, but there is still little information on the role of these interactions in freshwater ecosystems especially in the context of cyanobacterial growth and bloom development. We hypothesized that MOB could be an alternative C source to support cyanobacterial growth in freshwater systems. We detected low δ^13^C values in cyanobacterial blooms (the lowest detected value −59.97‰ for *Planktothrix rubescens*) what could be the result of the use of methane-derived carbon by cyanobacteria and/or MOB attached to their cells. We further proved the presence of metabolically active MOB on cyanobacterial filaments using the fluorescein isothiocyanate (FITC) based activity assay. The PCR results also proved the presence of the *pmoA* gene in several non-axenic cultures of cyanobacteria. Finally, experiments comprising the co-culture of the cyanobacterium *Aphanizomenon gracile* with the methanotroph *Methylosinus sporium* proved that cyanobacterial growth was significantly improved in the presence of MOB, presumably through utilizing CO_2_ released by MOB. On the other hand, ^13^C-CH_4_ labeled incubations showed the uptake and assimilation of MOB-derived metabolites by the cyanobacterium. We also observed a higher growth of MOB in the presence of cyanobacteria under a higher irradiance regime, then when grown alone, underpinning the bidirectional influence with as of yet unknown environmental consequences.

## Introduction

Freshwater ecosystems are estimated to be among the largest natural sources of atmospheric methane (Kirschke et al., 2013; Saunois et al., 2020; Rosentreter et al., 2021), a potent greenhouse gas of which atmospheric concentrations may increase due to feedback mechanisms as the result of global warming (Marotta et al., 2014). However, diffusive lake methane fluxes are mostly (30–99%, Bastviken et al., 2008) mitigated by aerobic methane-oxidizing bacteria (MOB), mostly belonging to Alpha- and Gammaproteobacteria (Bodelier et al., 2019) using methane for energy generation and cellular carbon. In this way, biogenic CH_4_ can subsidize the pelagic food web as an alternative energy and carbon source (Bastviken et al., 2003; Deines et al., 2007; Jones and Grey, 2011; Agasild et al., 2014) by predation of MOB by protozoa and metazoa, who transfer it into the pelagic food web (Bastviken et al., 2003; Kankaala et al., 2007; Schilder et al., 2017). Aerobic methanotrophs can also thrive under oxygen-deficient conditions, which are common in eutrophic and hypertrophic stratified lakes (Yang et al., 2019; Mayr et al., 2020). This can be explained by their versatility in using other electron acceptors, e.g., nitrite/nitrate, humic acids, or ferric ions (Kits et al., 2015; Oswald et al., 2017; Naqvi et al., 2018; van Grinsven et al., 2020, 2021). However, the near complete consumption of methane was observed in anoxic layers of a stratified lake when algae could perform photosynthesis and supply MOB with oxygen (Milucka et al., 2015; Oswald et al., 2015). A similar phenomenon was described in the leaves and stems of aquatic plants that supported the methanotrophic activity (Yoshida et al., 2014). Such cohabitation of MOB and photosynthetically active organisms has already been investigated for potential use in effective methane-removing biotechnologies, in which algae provide MOB with oxygen and MOB produce CO_2_ in return (van der Ha et al., 2011; Badr et al., 2019). However, this exchange of substrates between MOB and photosynthetic phytoplankton has mostly been demonstrated in reactor/biotechnological settings. An *in situ* example of this interkingdom methane-derived carbon exchange has been described in peat bogs, where methanotrophs were present in hyaline cells and on stems and leaves of *Sphagnum* and rapidly oxidized methane to CO_2_, which constituted a significant source of carbon (10–15%) for the peat moss (Raghoebarsing et al., 2005). Organic compounds released by methanotrophs such as methanol, formate, acetate, and other metabolites can potentially support a broad range of microbes (Chistoserdova and Kalyuzhnaya, 2018). In this respect, cyanobacteria can grow mixotrophically (Schmetterer and Flores, 1988; Stebegg et al., 2019), while genes related to methane oxidation have been detected in the “cyanosphere” of two blooming cyanobacteria (Pascault et al., 2021), indicating the possibility of metabolite exchange between these bacterial guilds.

Carbon dioxide is an important factor influencing the structure of phytoplankton communities (Shapiro, 1997). Primary production increases with trophy (Peters, 1986), and thus eutrophic and hypertrophic waters may be undersaturated with CO_2_ due to high phytoplankton productivity (Finlay et al., 2009; Lazzarino et al., 2009). Dense blooms often deplete dissolved CO_2_ below the atmospheric equilibrium (Talling, 1976; Maberly, 2008; Balmer and Downing, 2011). However, in deep, eutrophic and stratified lakes, the meta- and hypolimnion can be rich in CO_2_ and HCO_3_ as the result of biological decomposition and chemical reactions (Heaney et al., 1986). The oxidation of methane may account for a high proportion of excess inorganic carbon accumulation in the hypolimnion of stratified lakes (Houser et al., 2003), turning methane-derived carbon into a more relevant carbon source than photosynthetically produced carbon under more eutrophic conditions (Schilder et al., 2017). However, a quantitative prediction of the feedback between phytoplankton growth, methane oxidation, and CO_2_ drawdown in aquatic ecosystems has garnered little attention. This is surprising since CO_2_ may perform a crucial role in the competition among phytoplankton species, including harmful cyanobacteria that threaten the water quality of many eutrophic and hypertrophic lakes and cause severe ecological and economic damage worldwide. Cyanobacteria with high-flux bicarbonate uptake systems can benefit from elevated CO_2_ levels (Ji et al., 2017). Although the light conditions in the meta- and hypolimnion can be poor due to high primary production, cyanobacteria are adapted to them. For example, *Aphanizomenon* has a competitive advantage in light-limited conditions due to its affinity for light (De Nobel et al., 1998). Low light intensity and high nutrient concentrations, in combination with CO_2_ provided by methane oxidation in the hypo- and metalimnion, may provide cyanobacteria with an advantage over other phytoplankton, e.g., green algae (Shapiro, 1997). Moreover, some cyanobacteria can regulate their vertical distribution (buoyancy) and move up and down the water column to seek optimal light and nutrient conditions (Walsby and Booker, 1980; Walsby et al., 2004; Carey et al., 2012). In this way, cyanobacteria can gain extra benefits by positioning themselves closer to the chemocline, where in stratified lakes, the majority of methane is oxidized by MOB (Schubert et al., 2010).

Biogenic methane has the lowest isotopic carbon ratio compared to other natural sources because it is extremely depleted in ^13^C (Whiticar, 1999). Organisms assimilating this ^13^C-depleted carbon directly (e.g., MOB) or indirectly (e.g., phyto- or zooplankton) will have more negative δ^13^C values of their cellular carbon compared to other components of the food chain. Cyanobacteria exhibit variable δ^13^C values, which are quite often below −30.6‰ (Vuorio et al., 2006; Agasild et al., 2019). These low δ^13^C values are much lower than isotopic signatures usually found in inorganic carbon sources (Rinta et al., 2015) that can be utilized during photosynthesis. Previously reported CH_4_ oxidation linked to photosynthetic activity and overlapping niches motivated us to explore the possibility of associations occurring between MOB and cyanobacteria. We suspected that there are associations between cyanobacteria and MOB, which would result in the low observed δ^13^C values, and the latter could be an alternative carbon source supporting cyanobacteria growth.

To test this hypothesis, we analyzed filamentous cyanobacteria collected in the field, for the presence of metabolically active MOB dwelling in the cyanosphere or attached to cyanobacteria. We also tested several cyanobacterial laboratory strains for the presence of particulate methane monooxygenase gene *pmoA*.

We also hypothesized that carbon derived from CH_4_ oxidation by MOB subsidizes photoautotrophs, such as cyanobacteria in stratified lakes. We tested this hypothesis under laboratory conditions, first testing the possibility of cyanobacteria growth in the presence of methane and methanotrophic bacteria without external input of CO_2_, and second, testing the ^13^C transfer from CH_4_ to cyanobacteria. Since we had no access to axenic cultures of cyanobacteria and there are known examples of methanogens and MOB associations with phytoplankton (Grossart et al., 2011; Mulhollem et al., 2016; Samad et al., 2020; Li et al., 2021), we decided to test the possibility of carbon transfer via MOB metabolites utilized by other organisms that later release CO_2_ or organic substances. We also tested the influence of light conditions on the overall performance of the consortium. There are known examples from aquatic ecosystems showing that light intensities as low as 4.1 μmol photons m^–2^ s^–1^ can significantly decrease methane oxidation (Murase and Sugimoto, 2005), and laboratory experiments showed 90% growth inhibition for *Methylosinus* and enriched cultures of natural methanotroph communities (Dumestre et al., 1999). We expected that stronger light conditions would inhibit the growth of light-sensitive MOB and cyanobacteria and reduce the overall performance of both organisms (e.g., CH_4_ oxidation and cyanobacteria production), while low light intensity would enhance the overall performance of both organisms. Here, in this study, we used a model system of the filamentous cyanobacterium *Aphanizomenon gracile* (strain SAG 31.79), common to eutrophic lakes, and the methane oxidizing bacterium *Methylosinus sporium* (NIOO collection, strain L17-3), isolated from a freshwater lake.

## Materials and Methods

### Sampling and Isotope Analyses of Lake Phytoplankton and Lake Gases

The samples for isotope analyses of lake phytoplankton were taken from the epi-, meta-, and hypolimnion during blooms of either algae or cyanobacteria in 2016–2018 from Budzisławskie, Łagowskie, Licheńskie, Łódzko-Dymaczewskie, Mikorzyńskie, and Trześniowskie lakes using a 5 L Uwitec water sampler. Detailed information on the sampled lakes is provided in Supplementary Table 1. To separate phytoplankton from other organisms, samples were sieved through plankton nets of varying mesh size (30, 100, and 250 μm). This allowed most of the filamentous phytoplankton forms to be separated and condensed. In the case of some coccal cyanobacteria, we used separation flasks where light was applied from the bottom attracting most zooplankters, and positive buoyancy caused scum formation, which was then collected with a Pasteur pipette. Only samples that contained > 90% of a single phytoplankton taxon were chosen for analysis. The separated material was then filtered on a precombusted (500°C, 4 h) GF/F Whatman filter, freeze-dried and stored in a desiccator until further analyses. The water sampled for CH_4_ and CO_2_ analyses was alkalized with KOH and the water sampled for dissolved inorganic carbon (DIC) analysis was acidified with HCl and kept in 60 mL serum bottles stoppered with butyl septa and crimped.

Stable isotope ratios (^13^C/^12^C) in the phytoplankton samples were determined using an elemental analyzer (Flash 2000, Thermo Fisher Scientific, Waltham, MA, United States) coupled via a Conflo IV to an isotope ratio mass spectrometer (IRMS, Thermo Deltra XP advantage, Thermo Fisher Scientific, Waltham, MA, United States). The separated phytoplankton were sorted into tin cups and folded into compressed balls. These were measured against reference standards of carbon (Supplementary Table 2) as described in Werner and Brand (2001) and modified according to Paul et al. (2007).

Stable carbon isotope measurements in lake gases were carried out using a Trace GC−ultra gas chromatograph attached to a Thermo Fisher Scientific Delta−V isotope ratio mass spectrometer (IRMS) via a combustion and high temperature reduction interface, respectively (GC Isolink, Thermo Fisher Scientific). The GC coupled to the IRMS was equipped with a 25 m PoraPlot capillary column (i.d. 0.32 mm; 0.10 μm film thickness). The oven temperature was programmed from 30 to 180°C at a rate of 5°C/min followed by an isothermal period of 5 min. Helium was used as the carrier gas. For calibration, a CO_2_ standard gas was injected at the beginning and at the end of each analysis. Analytical reproducibility was controlled by repeated measurements of the calibration gas.

The δ^13^C isotope ratios of the samples were expressed as the relative difference in the isotope ratio between the sample and the international reference standard in parts per thousand:


$$\delta \,\text{C}\,\left(‰\right)=\left({\text{R}}_{\text{sample}}/{\text{R}}_{\text{standard}}-1\right)\times 1000,$$

where δC is δ^13^C and R is the isotope ratio ^13^C/^12^C in the sample and in the standard, respectively. The R_standard_ to which the samples were compared to was Vienna PeeDee Belemnite (V-PDB).

### Detecting Metabolically Active Methane-Oxidizing Bacteria in Freshwater Systems

Samples for the detection of active MOB were taken from Licheńskie and Łódzko-Dymaczewskie lakes located in the Wielkopolska region of Poland (details about the lakes and their sampling are provided in Supplementary Table 1).

In the laboratory, a 100 mL water sample from each depth, lake and season was filtered through 0.2 μm polycarbonate filters. Filters were kept at 7.5 and 18.0°C, corresponding to the lake water temperatures in fall and summer, respectively, and stored in Petri dishes (ø2.5 cm) containing 2 mL of 50 mM NaHPO_4_ buffer (pH 7.5) for 30 min to 1 h in the dark. In the summer sampling campaign, samples below the oxycline were filtered in an anaerobic chamber under a N_2_ gas atmosphere.

In order to label the active MOB we used the “suicide substrate” assay, where a sample is incubated with a mixture of fluorescein isothiocyanate (FITC) and propargylamine. These two compounds react and form “fluorescein thiocarbamoyl-propargylamine” (FTCP) which leaves an acetylene functional group attached to a fluorescein label (McTavish et al., 1993). Acetylene (C_2_H_2_) acts as a suicide substrate for both monooxygenase enzymatic forms, soluble and particulate (Prior and Dalton, 1985), and also can inhibit the ammonia monooxygenase enzyme in nitrifiers (McTavish et al., 1993). Labeling of active MOB was based on an existing protocol with minor modifications (Pratscher et al., 2018). In summary, the filtered biomass was incubated for 30 min with a fresh solution of fluorescein thiocarbamoylpropargylamine (FTCP), rinsed with PBS 1X, treated with an antifadent (CitiFluor^®^) and finally detached from the filter. Further details on the labeling are found in Supplementary Appendix 2: FTCP labeling of active MOB.

The FTCP-labeled biomass was analyzed and sorted with an influx cell sorter (BD Biosciences, Franklin Lakes, New Jersey, United States) equipped with 200 mW 488 nm (blue) and 640 nm 120 mW (red) lasers and a 70 μm nozzle. Samples were first vortexed for 3 s and then sonicated with an ultrasonic probe for 30 s (20 kHz, 20 W) to enhance cell dispersion. The first sorting decision was made based on the FTCP fluorescent signal (ex488 nm/em580 nm) related to metabolically active MOB. Subsequent division was made based on the fluorescence of the natural pigments present in the phytoplankton cells: chlorophyll *a* (Chl *a*) for algae (ex488 nm/em692 nm) and Phycocyanin (Phy) for cyanobacteria (ex640 nm/em670 nm) (Frenken et al., 2020). Based on this, 3 “MOB groups” were sorted: free-living (FITC + ; Chl*a−*; Phy−), MOB attached to algae (FITC+; Chl*a*+*;* Phy−) and MOB attached to cyanobacteria (FITC+, Chl *a*+; Phy+). The trigger was set on the side scatter signal, and the event rates ranged from 100 to 1,000 events s^–1^ in 1-drop purity mode.

In order to apply Confocal Laser Scanning Microscopy (CLSM) to visualize active MOB-like bacteria attached to cyanobacteria and free-living the “suicide substrate” was performed on water samples from the epilimnion in Licheńskie lake. Immersion objective Plan Apo 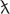 100X oil (1.45 NA) was used. Image acquisition from 5 z-stacks (0.25 μm/each) was performed using a Nikon A1 plus with NIS Elements AR software (Nikon, Tokyo, Japan). Wavelengths used for FITC, DAPI, and Chl a (autofluorescence) were: 488 ex/500–550 em., 402 nm ex,/425–475 nm em, and 488 ex./663–738 em., respectively. Samples were mounted on glass slides embedded in CitiFluor^®^ (AgarScientific).

### Detection of the Particulate Methane Monooxygenase Gene in Non-axenic Cultures of Cyanobacteria

Strains of *Aphanizomenon gracile* Lemmermann (AMU-DH-1, AMU-DH-7, CCALA8, SAG 31.79), *Aphanizomenon klebahni* Elenkin ex Pechar (AMU-DH-36), and *Planktothrix agardhii* (Gomont) Anagnostidis and Komárek (AMU-DH-42) were used to search for the *pmoA* gene. Some of the strains were obtained from the Culture Collection of Autotrophic Organisms of the Institute of Botany of the Academy of Science of the Czech Republic (CCALA8) and the Culture Collection of Algae at Göttingen University in Germany (SAG 31.79). The AMU strains were isolated from different Polish waterbodies using the procedure described in Zapomělová et al. (2007) and deposited in the Culture Collection at the Department of Hydrobiology, Adam Mickiewicz University in Poland. In brief, the isolation technique allows transferring a single filament out of a field sample, and such filament is transferred to sterilized media. The technique allows establishing a cyanobacterial strain of one genotype, however, some bacterial impurities may remain, especially when they are attached to a filament. Strains were identified according to the morphological criteria provided by Komárek and Anagnostidis (2007) and Komárek (2013). Stock cultures were maintained in glass Erlenmeyer flasks (Kavalier Glass, Sázava, Czech Republic) filled with 150 mL of WC medium (Guillard and Lorenzen, 1972) with excess concentrations of phosphorous and nitrogen for ∼20 days (time depended on the strain) from inoculation. They were maintained in a walk-in phytotron chamber (Conviron, Winnipeg, Canada) with a PAR irradiance of 50 μmol photons m^–2^ s^–1^ measured with a light meter LI-192 quantum sensor Li-COR (Bio-Sciences, Lincoln, United Kingdom), a photoperiod of 16:8 h light: dark cycle, and a temperature of 20 ± 0.5°C.

Amplicons of the particulate methane monooxygenase gene (*pmoA*) were produced from DNA extracted from cyanobacterial cultures using a “Genomic Mini AX Bacteria + “ kit (A&A Biotechnology, Poland) and a two-step PCR protocol. The first round of PCR amplification consisted of 35 cycles in which *pmoA*-targeted primers A189 and A682 (Holmes et al., 1995) were used. The thermal cycle profile consisted of initial denaturation for 5 min at 94°C, followed by 35 cycles of 1 min at 94°C, 1 min at 56°C, and 1 min at 72°C (5 min at 72°C for the last cycle). Reaction volumes of the first round were 25 μL and contained 2.5 μL of 5 pMoles of both reverse and forward primers, 0.125 μL of FastStart™ High Fidelity PCR System (reference 03553400001, Sigma Aldrich), and 1 μL of extracted genomic DNA.

Before the second round of PCR amplification, the PCR products from the first step were diluted 50-fold. The thermal cycle consisted of 5 min at 94°C, followed by 30 cycles of 30 s at 94°C, 30 s at 56°C, and 45 s at 72°C (5 min at 72°C for the last cycle). Primers A189 and mb661 (Costello and Lidstrom, 1999) were used. Reaction volumes of the second round were 50 μL. All PCR amplifications were performed in a BioRad c1000 Touch thermal cycler (Bio-Rad, Hercules, United States). The resulting PCR product was placed on a 1.5% agarose gel, and the band of the expected size was cut from the gel and purified with a QIAquick PCR Purification Kit (Qiagen). Products were Sanger sequenced by Macrogen Europe, and the most similar sequence was obtained using NCBI BLASTn.

### Cyanobacteria-Methane-Oxidizing Bacteria Interaction Experiments

#### Experimental Organisms

Two laboratory experiments were conducted using a planktonic photoautotroph and a methanotroph to test cyanobacteria-MOB interactions (see Supplementary Figure 1 depicting the experimental design). We used the non-axenic filamentous nostocalean cyanobacterium *A. gracile* (strain SAG 31.79) as a model of the photoautotroph. The mass culture of this strain was grown in a similar manner as the stock cultures used for detection of the *pmoA* gene.

As a model of MOB, we used the *Methylosinus sporium* strain, which was isolated from the pelagic zone of Lake Licheńskie in Poland in 2017 (NIOO collection, strain L17-3). One milliliter of lake water from Lake Licheńskie was put into 120 mL flasks, and 19 mL of five-times diluted nitrate mineral salt medium (M2; Dedysh et al., 1998) was added to it. Flasks were capped with gray rubber stoppers (Z166065 Sigma–Aldrich, St Louis, MO, United States), and pure methane was added to them (20% of CH_4_ in air v/v). The enrichments were incubated for 4 weeks at 20°C in the dark. After growth was observed, the enrichments were plated onto solid M2 medium containing 1.5% agarose. Plates were incubated in airtight jars supplemented with ambient air and 20% methane. Selected colonies were streaked onto fresh plates to obtain single colonies. Colonies were identified by Sanger sequencing (Macrogen Europe BV) of PCR products using primers A189 and A682 (Holmes et al., 1995). *M. sporium* L17-3 was further maintained in NMS media (Whittenbury et al., 1970) at 20°C in darkness and in gas-tight serum vials with crimp-sealed butyl stoppers with an air:methane ratio of ∼1:2. Bacteria belonging to the genus *Methylosinus* sp. are type II methanotrophs that are abundant in the stratified water column of the eutrophic Lake Licheńskie (Supplementary Figure 2). All manipulations of the cultures (e.g., inoculating cultures, fresh medium supply, or sample collection for observation) were performed in a laminar flow cabinet.

#### Preparing Stocks of Experimental Organisms

Before the start of the experiment, the mass culture of *Methylosinus* sp. was centrifuged (Rotina 380R centrifuge, Germany) with subsequent resuspension of the pellet in sterile NMS medium. The initial concentration of the stock was estimated by absorbance measurement at 600 nm (Metertech, SP-830 Plus, Taipei, Taiwan), and the carbon content was calculated using a previously established absorbance-carbon regression equation. A similar procedure was applied to the *A. gracile* culture, and the absorbance at 750 nm of the stock was used in the absorbance-carbon regression equation. Based on the carbon content in both stocks, the concentration of organisms in the inoculum was estimated.

#### Growth of the *Aphanizomenon*—*Methylosinus* Co-culture

The first experiment consisted of 6 treatments: *Methylosinus* alone (Met), *Aphanizomenon* alone (Aph), and a combination of both organisms (AphMet). The designed consortia were split randomly into two groups cultured under different light conditions: high light (HL, 105 μmol s^–1^ m^–2^ PAR) and low light (LL, 15 μmol s^–1^ m^–2^ PAR) intensities. Each treatment was replicated 5 times, resulting in 30 replicates in total. The 125 mL serum bottles were filled with equal volume aliquots of stock culture and filled with NMS medium enriched in P (8.71 g of K_2_HPO_4_ L^–1^) up to 35 mL. The addition of extra phosphorous ensured no competition for this element and allowed for a better growth of *Aphanizomenon* on NMS media, which were not designed for this organism. After mixing, quantitative samples of the organisms were taken (1 mL for *M. sporium* counts using qPCR, 1 mL for *A. gracile* counts using flow cytometry and morphological analyses, 0.3 mL for absorbance measurements), and then the bottles were sealed with crimp sealed butyl stoppers. A 30 mL sterile syringe equipped with a membrane disc filter (0.2 μm) was used to suck 30 mL of air out of the bottle and then refill it with 20 mL of methane (purity 5.5., Linde AG). Gas samples were taken after mixing the contents to obtain initial concentrations. The bottles were incubated in walk-in phytotrons (Conviron), and after 12 days, the experiment was terminated. The gas and organisms were sampled once more at the conclusion of the experiment.

#### Gas Analyses in the *Aphanizomenon*—*Methylosinus* Co-culture

CH_4_ and CO_2_ concentrations in samples stored in exetainers were analyzed with a Trace GC Ultra (Thermo Fisher Scientific, Waltham, MA, United States) using an HP-PLOT/Q TCD detector and a capillary column (30 m long, 0.53 mm of internal diameter). Helium was used as the carrier gas. The temperature program of the column was as follows: isotherm at 33°C for 7.5 min, dispenser temperature of 240°C, and TCD detector temperature of 200°C. The change in gas concentrations was expressed as the difference in the gas concentration between the first and last day of the experiment.

#### Assessing the Biovolume of Cyanobacteria

During the experiment, cyanobacterial samples (1 mL volume) were collected quantitively from cultures on day 6 of the experiment, and they were immediately preserved with Lugol’s iodine solution and used for microscopic analyses of trichome morphometry (thickness and length). The thickness of 10 and the length of 100 randomly selected trichomes were measured in each sample using an Axioskop 2 mot plus light microscope (Carl Zeiss AG, Oberkochen, Germany) equipped with a Jenoptik ProgRes Speed Xtcore3 digital camera and ProgRes image capture software (Jenoptik Optical Systems, Jena, Germany). Trichome thickness was measured in the middle part of the trichomes. Morphometric data were used to calculate the average trichome biovolume (mm^3^) following the formula for computing the volume of a cylinder, which corresponds to the geometric shape of *A. gracile* (Vadrucci et al., 2007). Trichome density in the samples was determined based on counting trichomes using a Beckman Coulter Cytomics FC 500 MPL flow cytometer (Beckman Coulter Life Sciences, Brea, CA, United States), and beads (C36950 Countbright Absolute Counting, Thermo Fisher Scientific, Waltham, MA, United States) were added as a counting reference (10 μL/1 mL of a sample). Trichome biovolume and density data were then used to calculate the biomass of *A. gracile* through the following formula:


$$B\,\left(m\,m^{3}\,m\,L^{-1}\right)=B\,i\,o\,v\,o\,l\,u\,m\,e\times D\,e\,n\,s\,i\,t\,y.$$

#### Abundance of *Methylosinus sporium*

Samples were transferred into sterile 2 mL Eppendorf tubes and centrifuged. The supernatant was discarded, and the pellet was used for DNA isolation with a Genomic Mini AX Bacteria + (mod.5) kit (A&A Biotechnology, Poland). The isolated and purified DNA was dissolved in 100 μL of Tris HCl, pH = 8.5, and frozen until further analyses.

Because degenerated primers II223 and II646 (Kolb et al., 2003) produced non-specific amplifications during qPCR, we designed new specific primers for the amplification of the *pmoA* gene from our strain of *Methylosinus sporium* (Supplementary Appendix 1 Primer design). qPCR was performed using a StepOne Plus Real-Time PCR System (Thermo Fisher Scientific, Applied Biosystems, Waltham, MA, United States) apparatus in 96-well plates. The optimal qPCR parameters were applied to a full-scale study in which each 15 μL reaction mixture contained 7.5 μL of 2x PowerUp SYBR Green Master Mix (Thermo Fisher Scientific, Applied Biosystems; PN A25778), 2 μL of DNA preparation, and 9 picomoles of each primer. The applied thermal cycling conditions consisted of an initial incubation at 50°C for 2 min and denaturation at 95°C for 2 min, followed by 45 cycles at 95°C for 15 s, 58°C for 15 s, and 60°C for 60 s, and finally of a melting step. In each qPCR assay, standard curves were prepared using six fourfold dilutions of the purified gDNA preparation from 98 to 25,000 haploid genome copies of *Methylosinus sporium* (using 241015 copies per ng as a conversion factor). Standards, DNA samples, and the no-template control (NTC) were analyzed in triplicate in each assay. The specificity of the products was validated by melting curve analyses. The NTCs produced no amplification in all qPCR assays. The absolute quantifications compared to standards were analyzed using SDS 2.3 software (Applied Biosystems). Details of the qPCR assay are listed in Supplementary Table 3.

#### ^13^C-CH_4_ Transfer in *Aphanizomenon*—*Methylosinus* Co-culture

During the second 2-stage experiment, we tested whether methane-derived carbon is transferred to cyanobacteria via CO_2_ (1st stage) or via exudates of the MOB (2nd stage) (Supplementary Figure 1). The first stage consisted of 3 treatments: 8 replicates of *Aphanizomenon* alone (Aph), 8 replicates of *Methylosinus* alone (Met) and 8 replicates of a mixture of both (AphMet). Furthermore, these were randomly split into 2 groups, where one group received ^13^C-labeled methane and the other one received unlabeled methane.

Fresh mass cultures of *Methylosinus* and *Aphanizomenon* were processed in a similar manner as in the previous experiment.

The ^13^C-transfer experiment was conducted in 1.2 L flasks, and *Methylosinus* and *Aphanizomenon* cultures were resuspended in NMS media (200 mL in each flask) and subsequently sealed with butyl stoppers. CH_4_ was added by removing 200 mL of air from the flask and replacing it with 200 mL of ^12^CH_4_ (purity 5.5., Linde). Half of the methane-containing replicates were spiked with 10 mL of ^13^CH_4_ (^13^C, 99 atom% ^13^C, Sigma–Aldrich), resulting in ∼5,000‰ ^13^C. The first stage was concluded after 9 days, at which point the media in all the flasks were centrifuged, and the pellets were used for PLFA analyses. The remaining supernatant was filtered through Whatman GF/F and nucleopore filters (0.2 μm) and used in the second stage of the experiment.

For the second stage of the experiment, with the aim of assessing whether labeled exuded metabolites of MOB are taken up by cyanobacteria, 100 mL of fresh and P-enriched NMS medium was mixed with 50 mL of old filtered media containing *Methylosinus* metabolites from the Met treatment with either ^12^C or ^13^C methane from stage 1. Aliquots of fresh *Aphanizomenon* culture were added. Bottles were sealed and this time no CH_4_ was added. At the end of the 2nd stage, the *Aphanizomenon* filaments were centrifuged to concentrate them, and the pellet was transferred to a 2 mL Eppendorf tube and freeze-dried before further analyses of the PLFA and isotope ratios.

#### PLFA Extraction and ^13^C-Enriched PLFA Identification

Extraction and identification of PLFAs as well as the carbon isotopic composition of fatty acid methyl esters of the extracted lipids were executed as described in Ho et al. (2019).

PLFAs were extracted from 0.5 to 4 mg freeze-dried filtered microbial biomass from both stages of the second experiment, following the procedure of Frostegård et al. (1993) and Hedlund (2002), based on the method of Bligh and Dyer (1959) and White et al. (1979). PLFA extraction was analyzed on a gas chromatograph (GC-FID, 7890A, Agilent Technologies, Delaware, United States) to determine the abundance of the PLFA biomarkers. Identification of FAMEs was based on comparing the retention index data generated by GC-FID/GC-mass spectrometry (GC-MS; Thermo Finnagan TRACE GC-MS system, Thermo Fisher Scientific, Waltham, MA, United States) analysis with known standards and analyzed reference samples as described previously (Bodelier et al., 2013; Henneberger et al., 2014). The δ^13^C value for each PLFA biomarker was determined by analyzing PLFA extractions on a Thermo Trace Ultra GC interfaced with a Thermo Scientific Delta V IRMS (Thermo Fisher Scientific, Waltham, MA, United States). For both GC analyses, an Agilent HP-5MS UI column (60 m, 0.25 mm id, 0.25 μm film thickness) was used. The δ^13^C PLFA of labeled and unlabeled control samples was used to calculate the excess amount of ^13^C in each PLFA biomarker (Boschker, 2004).

#### Statistical Analyses

When the assumptions of normality and homogeneity of variances were met, a two-way ANOVA was performed, and Tukey HSD was used for multiple comparisons. In the case of gas concentrations and absorbance, where organisms or their effects could not be separated, we created dummy variables combining treatments with cyanobacteria and bacteria. One outlier in the concentration of CO_2_ (ca. 2.5 times higher concentration than the next highest value in the whole dataset) was considered a technical error and removed from analyses. Because of the lack of homogeneity of variances in the case of biomass yield of the co-cultures and *A. gracile* treatments, the GLS function in the non-linear mixed effects (nlme) library (Pinheiro et al., 2021) was applied with a contrast analysis within the best model using the Benjamini-Hochberg adjustment. All analyses were performed with R version 4.0.0 (R Core Team, 2020). Figures were prepared using ggplot2 library (Wickham, 2016).

## Results

### Isotopic Signature (δ^13^C) of Cyanobacteria in the Lake Samples

**FIGURE 1:**
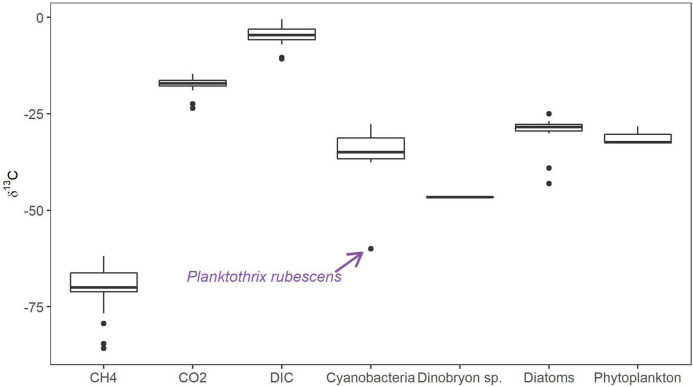
Median values of stable carbon isotope signatures (δ^13^C) of different phytoplankton groups and carbon sources in the sampled lakes during the 2017–2018 monitoring program. Bold horizontal line represents the median values, boxes represent 25th to 75th percentiles, and dots represent outliers.

During our monitoring program of Licheńskie, Mikorzyńskie, Budzisławskie, and Łódzko-Dymaczewskie Lakes in 2017 and 2018, we were able to isolate several phytoplankton taxa for the isotopic analyses of carbon (^13^C and ^12^C). Figure 1 presents the median values of δ^13^C for the main phytoplankton groups and dissolved inorganic carbon (DIC), CO_2_ and CH_4_. Values for the total phytoplankton community were similar to what has been reported in the literature; however, we observed several extremes in some members of the community. The lowest value was noted for the cyanobacterium *Planktothrix rubescens* in the meso-eutrophic Lake Budzisławskie (−59.97‰, Figure 1) sampled near the bottom of the lake. We also obtained *Dinobryon* sp. (Chrysophyta) with isotopic signatures below δ^13^C −40‰. The δ^13^C values of DIC and CO_2_ were much higher than those found in phytoplankton. Methane isotopic signatures had the lowest values out of all measured sources.

### Metabolically Active Methane-Oxidizing Bacteria: Free Living and Associated With Different Phytoplankton Components

**FIGURE 2:**
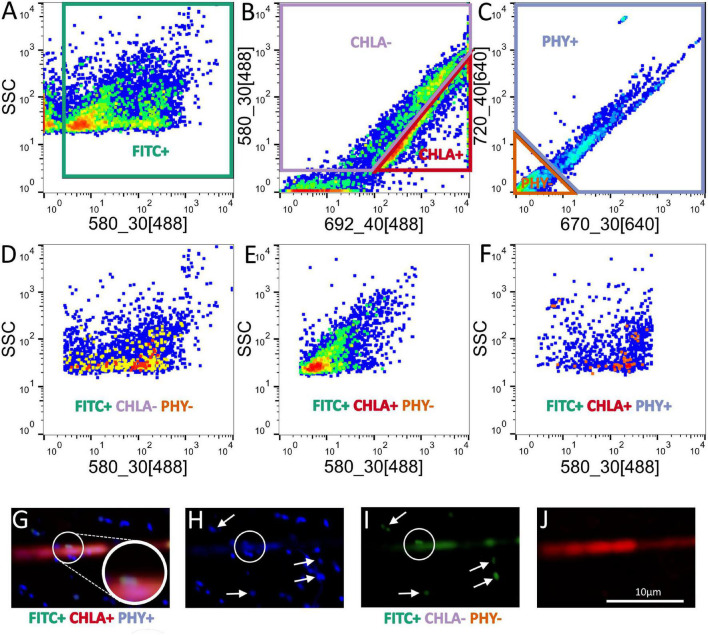
Dot plot with pseudo-color visualization of the sorted cell fractions and their gating strategy. **(A)** FITC gate in the side scatter vs. 580 nm (blue laser) dot plot (FITC+, gate in green). **(B)** Chlorophyll *a* gate in the 580 nm (blue laser) vs. 692 nm (blue laser) dot plot (CHLA+, gate in red). **(C)** Phycocyanin gate in the 720 nm (red laser) vs. 670 nm (red laser) dot plot (PHY+, gate in blue). Sorted cell fractions plotted (ssc vs. 580 nm (blue laser) are shown in the row below, gated with Boolean gating. **(D)** Free living MOB-like cells (gated as FITC+, CHLA–, PHY–). **(E)** Active MOB-like cells attached to algae (gated as FITC+, CHLA+, PHY–). **(F)** Active MOB-like cells attached to cyanobacteria (gated as FITC+, CHLA+, PHY+). **(G)** Three-channel depicts the presence of FITC+ cells (MOB-like cells, FITC fluorescence in green) attached to a filamentous cyanobacterium (Chlorophyll α autofluorescence in red). The inset depicts the presence of MOB-like cells (FITC +) on the filament. **(H)** DAPI stained cells. The circle shows the cells that were FITC+ on **(G,I)**. **(I)** Presence of FITC+ cells on the filament (circle), and the arrows show FITC + cells corresponding to free MOB-like cells. **(J)** Natural Chl a autofluorescence (red) from a cyanobacterium.

FTCP labeling coupled with FACS allowed us to retrieve 3 active MOB subpopulations from Licheńskie and Łódzko-Dymaczewskie Lakes (Figures 2A–F). These MOB were “attached” to algal and cyanobacterial cells as well as free-living cells (only the FTCP signal) (Figures 2G–J). At each depth, it was not always possible to retrieve the same number of events for each fraction. As depicted in Supplementary Figure 3, the success in retrieving MOB-related events varied according to the depth, fraction, and lake (almost 92% of all expected MOB-related events). The reason for this was the fact that in some cases, the material was completely exhausted preventing the analysis of other fractions. This was caused by the high sample volume demand of the stringent “one-drop purity” sort mode. For instance, MOB-Cyano fractions in Łódzko-Dymaczewskie Lake were not retrieved from 2 and 10 m (winter and summer, respectively) depths, and MOBCyano fractions could not be obtained from Licheńskie Lake at a depth of 10 m (both seasons).

### Detection of Methane-Oxidizing Bacteria in Non-axenic Cultures of Cyanobacteria

Supplementary Figure 4 shows PCR results of *pmoA* gene detection in genomic DNA extracted from cyanobacterial cultures. The nested PCR approach used resulted in the arrangement of additional bands, which made it necessary to cut out and sequence bands at the same or similar height as the positive control. Four out of the 6 bands returned a clean *pmoA* sequence most closely related to methanotrophs belonging to the family *Methylocystaceae* (Supplementary Table 4). The *pmoA* gene amplicons were obtained from strains of *A. gracile* (SAG 31.71, AMU-DH-1, AMU-DH-7, CCALA8). Two bands belonging to *A. klebahnii* (AMU DH-36) and *P. agardhii* (AMU-DH-42), which were at the same height as the positive control, returned bad sequences, suggesting that these were mixed PCR products of multiple MOB species. Assuming that bands at similar heights as the sequenced bands are derived from MOB, it can be concluded that most cyanobacterial cultures are colonized by MOB.

### Cyanobacteria and Methane-Oxidizing Bacteria Interaction Experiments

#### Yield of *Methylosinus*, *Aphanizomenon*, and Gases

**FIGURE 3:**
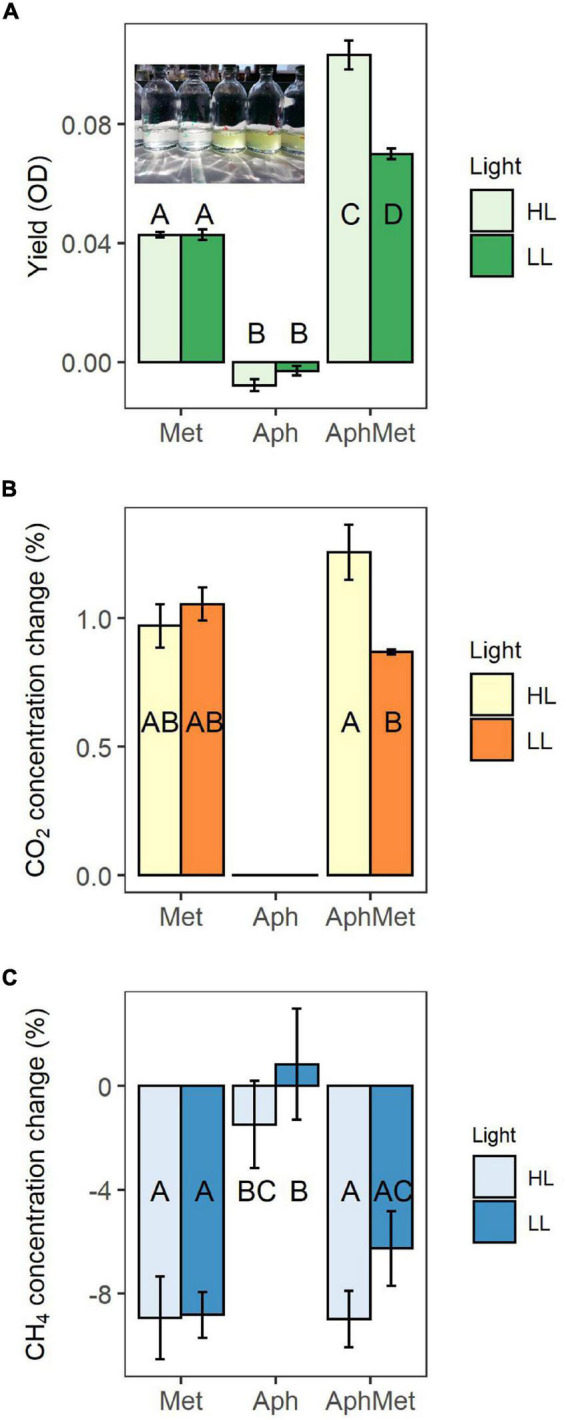
Yield of mono- and co-cultures expressed through optical density (OD), measured at 750 nm **(A)**, and changes in methane **(B)** and carbon dioxide **(C)** concentrations in these incubations. The inset picture represents cultures of *Aphanizomenon* only (left) and *Aphanizomenon* with *Methylosinus* (right) after 12 days. Bars represent the mean ± standard error (*n* = 5 for each treatment). Met, *Methylosinus*; Aph, *Aphanizomenon*; AphMet, mixture of *Aphanizomenon* and *Methylosinus*; HL, high light intensity; LL, low light intensity.

The first experiment explored the growth of *Aphanizomenon* and *Methylosinus* alone or in the co-culture in the presence of methane. We monitored the growth of the co-cultures via optical density measurements; thus, there was no distinction between MOB and cyanobacteria (Figure 3A). The co-culture (AphMet) grew better than *Methylosinus* alone regardless of the light conditions [GLS, *F*_(2, 24)_ = 708.8, *p* < 0.001], and *Aphanizomenon* alone even declined in either high light (HL) or low light (LL) intensity conditions. However, the yield of the co-culture (AphMet) interacted with the light conditions [GLS, *F*_(2, 24)_ = 22.3, *p* < 0.001] and was highest under HL conditions (Figure 3A), while growth was also promoted under LL intensity. *Methylosinus* significantly reduced the CH_4_ concentration in both the Met and AphMet treatments [ANOVA, *F*_(2, 24)_ = 18.376, *p* < 0.001], and there were no differences between the AphMet and Met treatments, nor was there a significant effect of light on methane removal [ANOVA, *F*_(1, 24)_ = 1.909, *p* > 0.05] (Figure 3B). The methane concentration did not change significantly in the Aph treatment (without *Methylosinus*). The CO_2_ concentration also differed among treatments, with no detectable CO_2_ production in incubations without *Methylosinus* (Aph treatment, Figure 3C). CO_2_ concentrations increased in treatments with *Methylosinus* (treatments AphMet and Met), and light also had a significant effect on its production. More CO_2_ was produced under HL in the presence of *Methylosinus* [ANOVA, *F*_(1, 15)_ = 4.566, *p* < 0.05]. There was also a significant interaction of light and the co-culture [ANOVA, *F*_(1, 15)_ = 9.301, *p* < 0.01], where more CO_2_ was produced under HL conditions when both *Methylosinus* and *Aphanizomenon* were present.

#### Growth of *Methylosinus*

**FIGURE 4:**
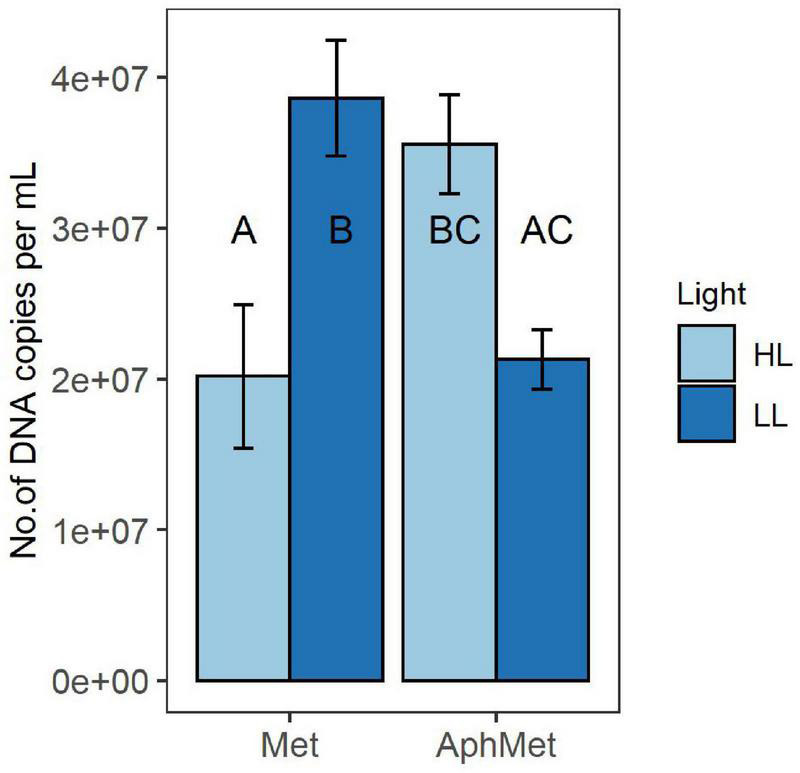
Yield of *Methylosinus* in the absence (Met) and presence (AphMet) of *Aphanizomenon gracile* under high light (HL) and low light (LL) conditions. The growth of *Methylosinus* is expressed as the number of *pmoA* gene copies per mL of medium. The same letters indicate homogenous groups. Bars represent the mean ± standard error (*n* = 5 for each treatment).

The growth of *Methylosinus* in the co-culture, measured as a change in the number of *pmoA* gene copies, was dependent on the presence of *Aphanizomenon* [2-way ANOVA *F*_(1, 16)_ = 5.623, *p* < 0.05) (Figure 4). *Methylosinus* reached the highest yield either alone under low light intensity conditions (Met and LL, Tukey HSD, *p* < 0.05) or under high light intensity but together with *Aphanizomenon* (AphMet and HL, Tukey HSD, *p* < 0.05). Simultaneously, *Methylosinus* had reduced yield when grown alone under HL intensity when compared to LL (Met, HL vs. LL, Tukey HSD, *p* < 0.05), but it was on similar level with *Methylosinus* grown with *Aphanizomenon* under LL intensity (Met, HL vs. AphMet, LL, Tukey HSD, *p* < 0.05).

#### Growth of *Aphanizomenon*

**FIGURE 5:**
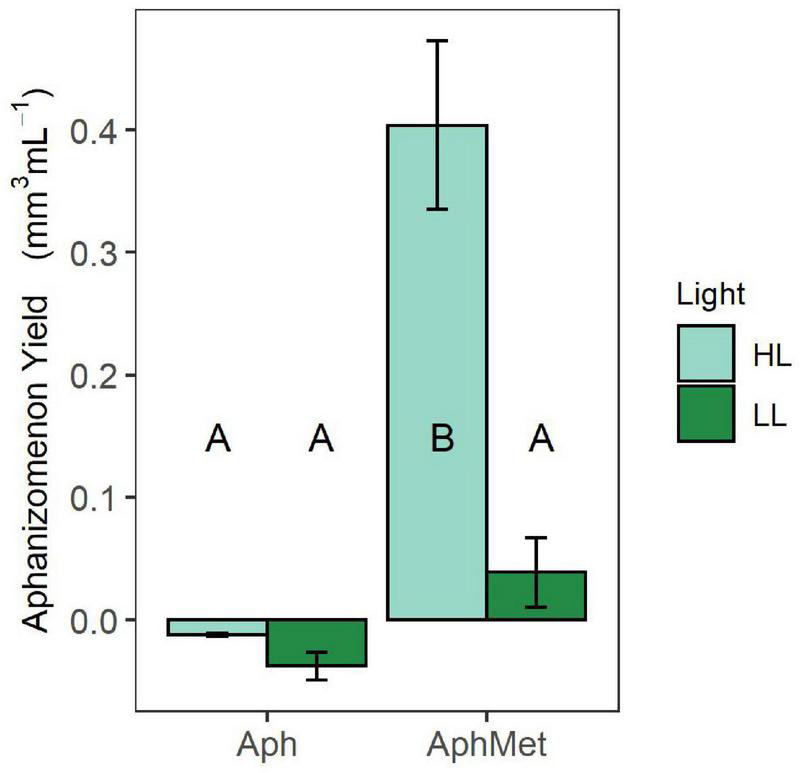
Yield of cyanobacteria during the experiment. Bars represent the means ± 1 standard error (*n* = 5 for each treatment).

The growth of *Aphanizomenon* occurred only in the presence of *Methylosinus* under high light intensity conditions (GLS model, interaction term, *t* = 4.522, *p* < 0.001) (Figure 5). All other incubations did not differ from each other (contrast analysis within the best model with Benjamini-Hochberg adjustment), while the biovolume of *Aphanizomenon* in the presence of *Methylosinus* under low intensity light conditions slightly increased or decreased in the absence of *Methylosinus.*

### ^13^C-CH_4_ Transfer in *Aphanizomenon—Methylosinus* Co-culture

**FIGURE 6:**
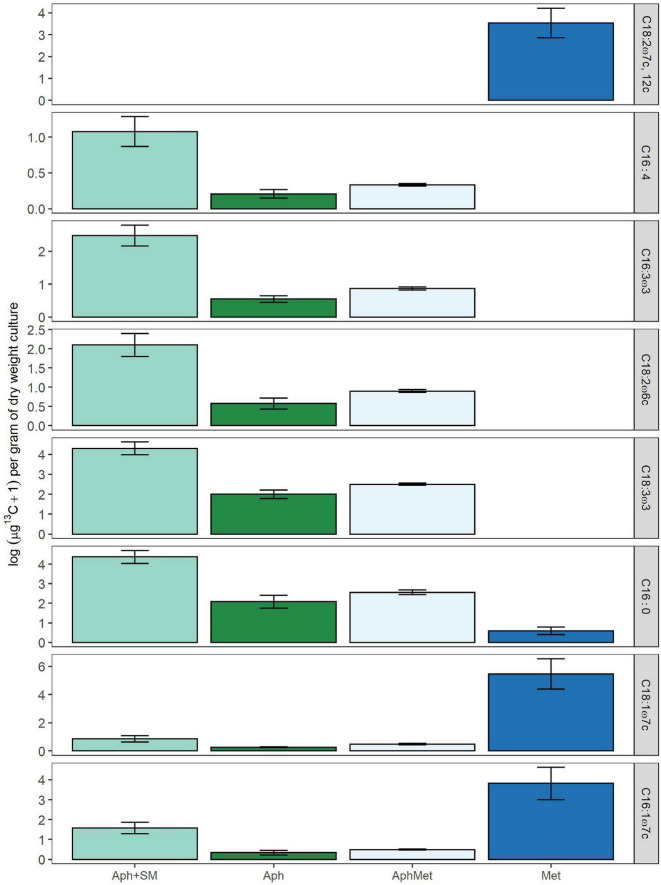
Log ^13^C content, as assimilated in individual PLFA molecules in mono- or co-cultures of *Aphanizomenon* (Aph) or *Methylosinus* (Met) expressed as μg of ^13^C per dry weight of total culture used in PLFA extraction. If ^13^C incorporation in this figure is higher than 0, the PLFA is enriched compared to control with ^12^C only. Aph + SM represents monocultures of *Aphanizomenon* with spent medium added originating from a *Methylosinus* monoculture grown with ^13^C methane. The δ^13^C PLFA of labeled and unlabeled control samples was used to calculate the excess amount of ^13^C in each PLFA biomarker. Bars represent means (*n* = 4) ± 1 SE.

From all the PLFA profiles of both *Methylosinus* and *Aphanizomenon* (Figure 6), we selected 1 PLFA that was exclusively detected in the methanotroph (i.e., C18:2ω7c, 12c), 4 which were detected only in the cyanobacterium (i.e., C16:1ω4, C16:3ω3, C18:2ω6c, C18:3ω3) and 3 which were detected in both (i.e., C16:0, C16:1ω7c, C18:1ω7c), which all had sufficiently high area values to ensure the correct determination of the carbon isotopic signature by subsequent GC-IRMS analyses. As expected, the highest ^13^C incorporation was found in the *Methylosinus* monoculture, having assimilated labeled methane with a starting isotopic enrichment of approximately 5,000‰ (0.5 atom% enrichment) (Figure 6). Surprisingly, PLFAs in *Aphanizomenon* monocultures were enriched in ^13^C derived from methane (Figure 6), which was roughly half of the amount in the mixed culture (13C APH/Mix ratio of 7 PLFAs = 0.56). The highest label incorporation by *Aphanizomenon* was observed in the cultures receiving spent filtered medium from the *Methylosinus* culture (Figure 6) which received no labeled CO_2_ but only labeled soluble exudates of the methanotrophs.

## Discussion

### Cyanobacteria—Methane-Oxidizing Bacteria Associations

Meta- and hypolimnion in eutrophic lakes can be rich in CH_4_, produced mainly by methanogenic Archaea in the sediments among other CH_4_ lacustrine sources (Günthel et al., 2019). The archaeal methanogenic pathway discriminates against ^13^C (Whiticar, 1999), thus organisms assimilating this ^13^C-depleted carbon directly (MOB) or indirectly (phytoplankton) should have more negative δ^13^C values of their cellular carbon compared to other components of the food chain. The oxidation of methane may account for a high proportion of excess inorganic carbon accumulation in the hypolimnion of stratified lakes (Houser et al., 2003). Additionally, MOB produce organic metabolites that can potentially support a broad range of microbes (Chistoserdova and Kalyuzhnaya, 2018). Cyanobacteria are frequently found in the oxic-anoxic zone where the majority of CH_4_ is oxidized to CO_2_ and with their high-flux bicarbonate uptake systems they can benefit from elevated CO_2_ levels (Ji et al., 2017) or even metabolites released by MOB.

We hypothesized that there are direct associations between cyanobacteria with MOB and/or that cyanobacteria utilize CO_2_ and metabolites from oxidized CH_4_ which would result in the low observed δ^13^C values. Using different yet supplementary approaches, we showed that MOB can be physically attached to and stimulate the growth of cyanobacteria via metabolic links. Thus, we tested phytoplankton collected in the field using FTCP labeling coupled with FACS, and we were able to distinguish active MOB cells that were either single (free-living) or in association with algae and cyanobacteria (Figure 2). Field reports have described the presence of MOB attached to phytoplankton using different techniques. For instance, alpha-proteobacterial MOB were detected using fluorescence *in situ* hybridization (FISH) in an algal cell (Milucka et al., 2015) or mRNA reads related to methane metabolism in the cyanosphere of bloom-forming cyanobacteria (Pascault et al., 2021). With respect to the technique applied to the study of the active MOB, it should be noted that FTCP could also label active nitrifiers, as this fluorophore can react with both ammonia monooxygenase and methane monooxygenase (McTavish et al., 1993). On the other hand, we were able to amplify the *pmoA* gene in several cyanobacterial isolates cultured under laboratory conditions. We could even detect *pmoA* sequences associated with non-axenic cyanobacterial cultures that have been cultivated under laboratory conditions for a long time. This suggests that methanotrophs survive and even propagate in cultures where no methane is added. However, cyanobacteria can produce small amounts of methane under oxic conditions, which is very likely connected to their photosynthetic activity (Bižić et al., 2020). Even though the amount of methane produced may be low, both *Methylocystis* spp. (Knief and Dunfield, 2005; Cai et al., 2016) and *Methylocapsa* spp. (Tveit et al., 2019) can thrive well under these conditions. Hence, the *Methylocystis*-type sequences we obtained from cyanobacterial isolates (Supplementary Table 4) match this idea of oligotrophic strains being able to survive in the cyanosphere. Additionally, other methanotrophs may be able to survive in the cyanosphere under natural conditions, where large amounts of methane may be formed during cyanobacterial blooms (Li et al., 2021). Another option for supporting MOB growth and activity may be hydrogen gas that is generated during nitrogen fixation by cyanobacteria (Lopes Pinto et al., 2002; Dutta et al., 2005). Some methanotrophs, such as *Methylocystis* possess hydrogenases, allowing them to generate energy from H_2_ oxidation (Hakobyan et al., 2020).

Initially, we conducted isotopic analyses of field samples containing phytoplankton and its selected members. Most of the δ^13^C values were in agreement with previous literature (e.g., Vuorio et al., 2006), but some taxa expressed lower isotopic ratios than expected, i.e., *P. rubescens* and *Dinobryon* sp. (Figure 1). Their low δ^13^C values could not originate only from DIC or CO_2_, as they were more positive (minimum values noted in Budzisławskie Lake were −5.8 and −16.4‰, respectively). High isotopic values for inorganic carbon sources may be the result of acetotrophic methanogenesis, where δ^13^C values of CO_2_ can be 30‰ higher than those in acetate used as a substrate in methane production (Conrad, 2005; Steinmann et al., 2008). The CO_2_ originating from acetotrophic methanogenesis does not exclude methane-derived inorganic carbon sources for phytoplankton, as observed in our experiments, but adds another source into the pool of inorganic carbon, and as a result, the mixture of different sources of CO_2_ has higher δ^13^C values. However, the differences in δ^13^C values of *Dinobryon* could be explained by the fact that they are mixotrophs and can feed on microorganisms, including MOB. For example, phototrophic flagellates can graze up to 79% of the total bacteria consumed (Sanders et al., 1989), and *Dinobryon* can selectively graze on Archaea (Ballen-Segura et al., 2017), which discriminate against ^13^C during methanogenesis. Cyanobacteria are also known for mixotrophy, but they can only incorporate dissolved organic carbon sources (Stebegg et al., 2019). This led us to think that there was another carbon source influencing the δ^13^C values of *P. rubescens* and possibly other phytoplankton groups. The qPCR revealed presence of high copy numbers of *pmoA* for Type II methanotrophs in Licheńskie and Łódzko-Dymaczewskie lakes (Supplementary Figure 2). These bacteria are capable of oxidizing dissolved CH_4_, and can add to the pool of CO_2_ available for cyanobacteria or release organic metabolites available for other microbes or mixotrophs. Our data do not allow for assigning the types of MOB attached to cells or designating the dissolved CO_2_ as the main source of carbon responsible for low δ^13^C values detected in the field samples. Hence, future work should determine which MOB species are active in the cyanosphere, what is their contribution to CH_4_ oxidation and carbon transfer to cyanobacteria, and which metabolic pathways are active in the cyanosphere dwelling MOB as compared to their free-living counterparts. It is also still not known what type of molecules are interchanged between MOB and cyanobacteria and how this interchange is regulated by environmental factors such as light or temperature. Further quantitative studies are required to assess the importance of different paths of carbon and energy flow, e.g., the uptake of metabolites by cyanobacteria from MOB.

### Cyanobacteria—Methane-Oxidizing Bacteria Interactions

To further investigate whether MOB can promote the growth of cyanobacteria, we conducted two laboratory experiments in which filamentous *A. gracile* grew in the presence of CH_4_ and *M. sporium* without external input of CO_2_, followed by ^13^C transfer from labeled CH_4_ to cyanobacteria. The results demonstrated that the methane-oxidizing bacterium *Methylosinus* subsidized *Aphanizomenon* with carbon. Moreover, there are two possible pathways of carbon transfer from MOB to cyanobacteria. One option is via direct transfer of CO_2_ produced during methane respiration by MOB, as shown in the first experiment. In these incubations where the cyanobacterium grew alone, the carbon dioxide concentrations decreased significantly, and the methane concentration was not affected (Figure 3). However, the cyanobacterium had the highest yield in the presence of *Methylosinus*, and there was a significant methane decrease with no CO_2_ limitation (Figure 3).

The second possible carbon pathway is the direct uptake of other released metabolites coming from the methanotrophs (Strong et al., 2015; Gilman et al., 2017) by the cyanobacterium or the possibility of carbon transfer via MOB metabolites utilized by other organisms attached to the non-axenic *Aphanizomenon*, which later release CO_2_ or organic substances. When exploring these pathways in the second experiment, we found that the *Aphanizomenon* monocultures incubated with only labeled methane were enriched in *^13^*C derived from methane at roughly half of the amount in the mixed culture. This can be explained by the fact that MOB are present in the cyanobacterial cultures, as we demonstrated by *pmoA* PCR, including the strain SAG 31.71 used in our experiments. Additionally, the highest ^13^C incorporation was found in *Aphanizomenon* alone and without CH_4_ when it was supplied with spent filtered medium from *Methylosinus* containing no ^13^CO_2_ but only labeled soluble exudates of the methanotrophs (Figure 6). The most plausible explanation is the presence of different bacteria feeding on labeled metabolites from *Methylosinus*, and producing labeled CO_2_ and/or *Aphanizomenon gracile* was able to assimilate organic carbon released by MOB. We have no data on the capability of *A. gracile* to assimilate organic carbon, but there are examples of it in other cyanobacteria, such as *Nostoc* sp., *Anabaena* sp., and *Synechococcus* sp. (Rippka et al., 1979; Schmetterer and Flores, 1988; Stuart et al., 2016; Stebegg et al., 2019). The influence of MOB cannot be excluded, but it was rather limited since in the second stage of the experiment with metabolites there was no CH_4_ in the atmosphere and thus no substrate for growth. Although cyanobacteria are capable of producing methane, e.g., *Nodularia spumigena*, and a phosphonate-degrading gene cluster was found in 28 sequenced cyanobacterial strains isolated from the Baltic Sea, no such genes were found in the *Aphanizomenon* genus (Teikari et al., 2018).

### Light Influence on Growth of *Aphanizomenon*, *Methylosinus* and Methane Consumption

Both organisms had higher yield when grown together, but this coexistence depended on light conditions. Contrary to our expectations, the consortium grown in high light intensity conditions had the highest CH_4_ consumption and CO_2_ and biomass production. *Methylosinus* alone grew better at low light than at high light intensity (Figure 4), and the whole consortium had a significantly lower yield at low light (Figures 4, 5). Moreover, the yield of *Aphanizomenon* in consortium under high light conditions was higher than that under low light intensity, and the yield of *Methylosinus* in consortium was similar to that in the treatment where *Methylosinus* grew alone under low light intensity conditions. Surprisingly, methane consumption in consortium treatment was similar at both light levels when compared to *Methylosinus* alone (Figure 3C). This suggests that the shading effect (i.e., self-shading by increasing biomass of organisms in a culture) had a role in it, and allowed for a better growth of *Aphanizomenon* and more efficient methane consumption by *Methylosinus* in high light intensity. The high light intensity condition in our experiment reached 105 μmol s^–1^ m^–2^ PAR, which is far above the known inhibiting light levels (Murase and Sugimoto, 2005), and it did not suppress CH_4_ consumption in the consortium nor in the *Methylosinus* alone treatment. However, when the *Methylosinus* was cultured alone the number of DNA copies was significantly lower under high light intensity when compared to low light intensity, which may suggest a change in the apparent cell-specific activity, i.e., increased metabolic activity but decreased abundance probably due to higher costs of growth in strong light stress. This response is different to known examples from aquatic ecosystems showing that light intensities as low as 4.1 μmol photons m^–2^ s^–1^ can significantly decrease methane oxidation (Murase and Sugimoto, 2005), and laboratory experiments showed 90% inhibition for *Methylosinus* and enriched cultures of natural methanotroph communities (Dumestre et al., 1999).

Another example of mutual support is the exchange of “oxygen for methane”—when both organisms are present, methane is oxidized by the methanotroph, which produces excess CO_2_, thereby avoiding carbon limitation of *Aphanizomenon*, and the full consortium had the highest yield as a result. Previous work (van der Ha et al., 2011; Milucka et al., 2015; Oswald et al., 2015) showed that methanotrophs can benefit from oxygen produced during photosynthesis, and Raghoebarsing et al. (2005) showed that methane-derived CO_2_ provides 10–15% of carbon for photosynthesis in *Sphagnum*.

However, our experiment started in a normal atmosphere enriched in CH_4_, with a surplus of oxygen. Thus, we cannot be certain whether *Aphanizomenon* supported or performed an important role in supplementing *Methylosinus* with oxygen.

### Synthesis

Species composition of phytoplankton depends on the magnitude of change in CO_2_ (Low-Décarie et al., 2011), and different requirements of various phytoplankton taxa for carbon influence interspecies competition. Dense blooms often deplete the dissolved CO_2_ in surface waters (Finlay et al., 2009; Lazzarino et al., 2009), however thermal stratification entraps methane and predicted climate warming will intensify this process. The methane is oxidized by MOB and may account for a high proportion of excess inorganic carbon accumulation in the hypolimnion of stratified lakes (Houser et al., 2003). Additionally, MOB produce organic metabolites that can potentially support a broad range of microbes (Chistoserdova and Kalyuzhnaya, 2018). The majority of CH_4_ is oxidized in chemocline, where cyanobacteria are frequently found. Thus, in eutrophic and hypertrophic lakes low light intensity and high nutrient concentrations, in combination with CO_2_ provided by methane oxidation in hypo- and metalimnion may provide buoyant cyanobacteria with an advantage over other phytoplankters (Shapiro, 1997). Our study showed that indeed cyanobacterial growth can be significantly stimulated by interaction with MOB in several ways, i.e., via direct supplement with CO_2_ and via metabolites. It is not clear which path is more relevant and further investigations are required, especially on the possibility of metabolite uptake by cyanobacteria. It is also not known whether cyanobacteria intake metabolites, e.g., from free-living bacteria, directly from bacteria living in cyanosphere or if they use CO_2_ released by other microbial organisms utilizing metabolites from MOB.

Whichever path is true we can say that MOB support the growth of cyanobacteria. The influence of methane-derived carbon dioxide and MOB on photosynthetic organisms, such as cyanobacteria, has been overlooked in aquatic ecology, but methane-derived carbon may perform an important role in the development of harmful cyanobacteria that threaten the water quality of many lakes. Only recently has the influence of phytoplankton biomass on greenhouse gas production been brought to attention (Bartosiewicz et al., 2021), and climate warming together with eutrophication strongly enhances the production of these gases. Our findings put the relative roles of MOB in the aquatic food web in a different perspective especially in the case of climate-induced increases in carbon and nutrient loading. MOB prevent the release of the diffusive CH_4_ fluxes from the water column (Bastviken et al., 2008), thus preventing release of a potent greenhouse gas to the atmosphere. Also, methane oxidation performs an important role in an aquatic microbial loop as predation on MOB by protozoa and zooplankton transfers energy and carbon to higher trophic levels (Bastviken et al., 2003; Kankaala et al., 2007; Schilder et al., 2017). Cyanobacteria often form intense blooms due to increasing eutrophication (Glibert et al., 2014) and global warming (Visser et al., 2016). Even though the larger the bloom the more CO_2_ can be sequestered, it should be noted that it eventually leads to hypoxia and in consequence the increase in CH_4_ production. As a consequence a positive feedback loop may enhance both cyanobacteria proliferation and methane production (Bartosiewicz et al., 2021; Bižić, 2021). In our study we have pointed to the overlooked alternative path that may have important consequences for lake ecosystem functioning.

## Data Availability Statement

The raw data supporting the conclusions of this article will be made available by the authors, without undue reservation.

## Author Contributions

SC: conceptualization, methodology, investigation, resources, data processing, formal analysis, visualization, writing—original draft, review response and editing, and funding acquisition. GP: investigation, data processing, visualization, and writing—original draft. MR, NH, MM-F, ŁP, and CR: investigation and writing—reviewing and editing. ŁW: investigation, resources, and writing—reviewing and editing. AK: investigation. SN-W: investigation and writing—reviewing. WN: methodology, investigation, and writing—reviewing and editing. PB: conceptualization, methodology, resources, data processing, writing—original draft, and supervision. All authors contributed to the article and approved the submitted version.

## Conflict of Interest

The authors declare that the research was conducted in the absence of any commercial or financial relationships that could be construed as a potential conflict of interest.

## Publisher’s Note

All claims expressed in this article are solely those of the authors and do not necessarily represent those of their affiliated organizations, or those of the publisher, the editors and the reviewers. Any product that may be evaluated in this article, or claim that may be made by its manufacturer, is not guaranteed or endorsed by the publisher.
