# Supplementary material for: Methane-Derived Carbon as a Driver for Cyanobacterial Growth
Source: Front Microbiol. 2022 Apr 1;13:837198. doi: 10.3389/fmicb.2022.837198 (PMC9010870; doi:10.3389/fmicb.2022.837198)
Supplement: Supplementary file 1 [file Data_Sheet_1.docx]

***Supplementary Material***

# Supplementary Data

**Table S.1** General information on lakes sampled in this study. The depths refer to the depths of the sampling stations.

| Lake | Type | Depth [m] | Surface [km^2^] | Trophy | Hypolimnion |
| --- | --- | --- | --- | --- | --- |
| Budzisławskie | Dimictic | 24 | 1.41 | Meso-eutrophic | Anoxic |
| Licheńskie | Dimictic/monomictic | 13 | 1.48 | Eutrophic | Anoxic |
| Mikorzyńskie | Dimictic | 30 | 2.52 | Eutrophic | Anoxic |
| Łódzko - Dymaczewskie | Dimictic | 11 | 1.21 | Hypertrophic | Anoxic |

For the Suicide substrate assay, samples were retrieved from every meter of the water column in each lake (0-10 m). Water was collected in a water sampler (Uwitec, Austria) and pre-filtered through a 100 µm mesh net. Water portions of one liter-water from each sampled depth, were kept in plastic bottles at 4°C until the moment of processing them.

**Table S.2** Reference standards of carbon as described in Werner and Brand 2001 and modified according to D. Paul et al 2007.

| Substance | Code | Procured from | δ^13^C | % C |
| --- | --- | --- | --- | --- |
| L-Glutamic Acid | USGS-40 | IAEA (Austria) | -26.39 | 40.8 |
| L-Glutamic Acid | USGS-41 | IAEA (Austria) | +37.63 |  |
| Sucrose | NIST-8542 | NIST (USA) | -10.45 |  |
| SULFANILAMIDE |  | Interscience (Breda) |  | 41.84 |
| ASPARTIC ACID |  | Interscience (Breda) |  | 36.09 |
| NICOTINAMIDE |  | Interscience (Breda) |  | 59.01 |

Paul, D., Skrzypek, G., Fórizs, I. (2007). Normalization of measured stable isotopic compositions to isotope reference scales – a review. Rapid Commun. Mass Spectrom. 21, 3006–3014. https://doi.org/https://doi.org/10.1002/rcm.3185

Werner, R.A., Brand, W.A. (2001). Referencing strategies and techniques in stable isotope ratio analysis. Rapid Commun. Mass Spectrom. 15, 501–519. ttps://doi.org/https://doi.org/10.1002/rcm.258

**Table S.3** Details information for each SYBR®Green qPCR assay. The desalted primers were purchased at Custom DNA Oligos Synthesis Services of ThermoFisher Scientific. *Cq variation given as SD at the endpoint of the linear dynamic range that corresponds to the lowest concentration in the linear interval of the calibration curve.

| qPCR assay name | Amplicon length | Forward primer | Reverse primer | Final concentration of each primer in qPCR reaction [µM] | Efficiency [%] | Intercept of standard curve | Slope of standard curve | r^2^ of standard curve | Error of standard curve | Cq variation at lowest limit (SD)* |
| --- | --- | --- | --- | --- | --- | --- | --- | --- | --- | --- |
| **Type2_140** | 140 | TGTCGGGCTCCTATGTGA | CAGATCAGCCAGAGACATCAG | 0.6 | 101.35416 | 35.978 | 3.2899 | 0.9993 | 0.023 | 0.135 |

**Appendix S.1 Primer design**

Specific primers pair were designed for amplification of the *pmoA* gene from *Methylosinus sporium*. First, fragments of the *pmoA* genes were amplified on gDNA template derived from *Methylosinus sporium* culture, using degenerated primers pair targets Methylocystaceae; type II group of methanotrophic bacteria (Kolb et al. 2003). The PCR was performed with Q5 High-Fidelity PCR Kit (New England Biolabs). The amplicon was directly sequenced bi-directionally (Big Dye Terminator v. 3.1 Cycle Sequencing kit, Applied Biosystems) on an ABI Prism 3130XL system (Applied Biosystems) for sequence identity. In that way resolved sequence was blasted to NCBI nucleotide database with the best hit to AJ459031, which was applied as template to design in silico new specific primers pair with use of Realtime PCR Tool available on Integrated DNA Technologies website. Primer pair specificity was verified by screening candidate primers against NCBI database limited to *Methylosinus sporium* in order to avoid primer pairs that could cause non-specific amplifications. Validation qPCR and DNA sequencing of its product confirmed specificity of primers. In order to ensure the most sensitive and most efficient qPCR assay, optimization experiment was performed by testing two qPCR parameters: annealing temperature and primer pair concentration, in interaction matrix of 3 different concentrations (300, 500 and 800 nM) with 3 different temperatures (54, 57 and 60 °C).

**Appendix S.2 FTCP labelling of active MOB**

A fresh fluorescein thiocarbamoylpropargylamine (FTCP) solution was prepared and left 2 h in the dark before its use. This solution contained 142 mM Fluorescein isothiocyanate (FITC) isomer I (Sigma) dissolved in DMSO (Sigma) and 400 mM of proparglyamine hydrochloride (Sigma) dissolved in 250 mM Na_2_CO_3_ in milliQ H_2_O. The FTCP solution had FITC and proparglyamine hydrochloride ratio of 7:3, v/v. Then, 200 µL of the FTCP solution was added to the samples for 1 h in the Petri dishes at RT in the dark. The incubation was ended by rinsing the filters carefully with PBS 1X to remove the excess of FTCP solution. Finally, filters were folded inside, embedded in some drops of antifadent mounting solution CitiFluor^TM^ AF1 (Electron Microscopy Sciences, USA) and kept at -20^o^C until further processing. All solutions were 0.2 µm filtered prior to their use.

Cell detachment from filters were carried out according to Couradeau et al., (2019) on the same day prior to the sorting. Filters were soaked in 5 mL of a 0.02% Tween® 20 (Sigma-Aldrich, ST Louis, MO, USA) Tween solution in PBS 1X and vortexed at maximum speed for 5 min. Finally, biomass was concentrated by centrifugation for 55 min at 500 g (Sigma 3-16KL, Germany). Four mL of the supernatant were carefully discarded by pipetting and the remaining volume was kept at 4^o^C and in darkness until their sorting.

Couradeau, E., Sasse, J., Goudeau, D. *et al.* Probing the active fraction of soil microbiomes using BONCAT-FACS. *Nat Commun* **10,**2770 (2019). https://doi.org/10.1038/s41467-019-10542-0

**Sorting strategy**

Flowsorter alignment was performed daily with Sphero 8 pk rainbow beads to a RCV below 3. Sample lines were flushed with sheath fluid (FACSflow, BD biosciences) at start up and between sorted samples. To achieve the highest purity, the sorting mode was set at the “one-drop purity” mode and sorted in separate 1-way sort sessions of 1- 40 minutes. Sort alignment was checked every hour with Accudrop beads (BD biosciences).


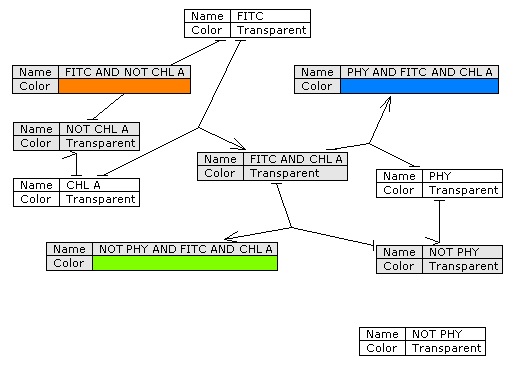


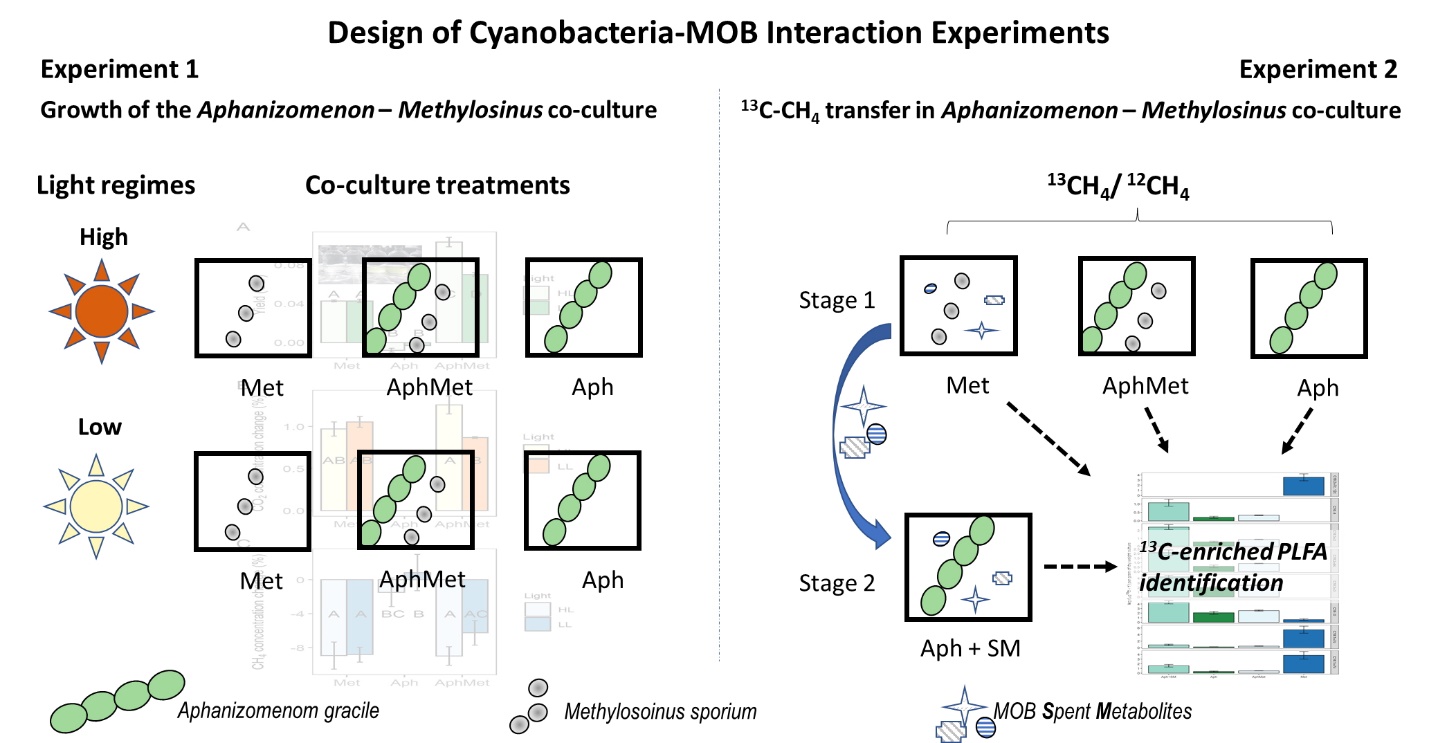


**Fig. S.1** Workflow of the interaction experiments in *Aphanizomenon-Methylosinus* co-cultures


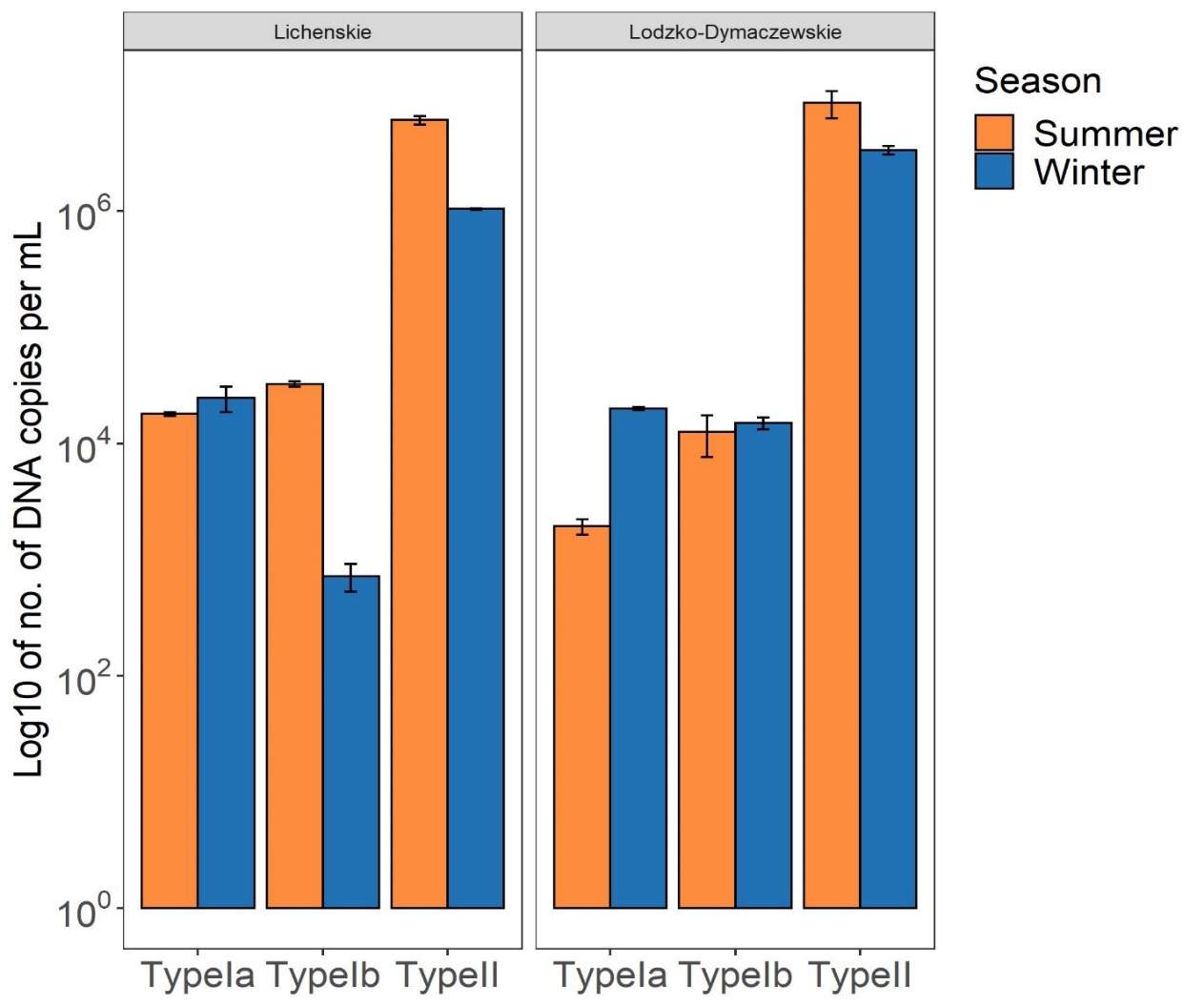


**Fig. S.2** Planktonic MOB abundances determined by qPCR. The bar plot depicts the average ± SD of the n copies target gene mL^-1^  filtered water of the aerobic MOB Type Ia, Ib and II in the thermocline of lakes Łódzko-Dymaczewskie and Licheńskie in September and same depths in November, 2019. Total genomic DNA was extracted using the DNeasy PowerSoil Kit (Qiagen, Venlo, The Netherlands), following manufacturer instructions. Water portions (100-120 mL) were filtered using 0.2-μm cellulose nitrate membrane filters (SartoriusTM) and half of the filter was used to do extraction. qPCR assays targeting MOB Type Ia, Ib and II were based on the quantitative *pmoA* method developed by Kolb et al 2003. DNA extract-sample-, non-template- control negative control-and standard curve, was done in duplicates. Standard curves were obtained from a 10-fold serial dilution of a known amount of plasmid DNA fragment from pure cultures representing the target gene (107–101 *pmoA* gene copies). Amplification efficiencies for Type Ia (77,8%, r2=0.995), Type Ib (87,9%, r2=0.998) and for Type II (87,4%, r2=0.999). Amplicon specificity was checked from the melting curve and by running samples on a 1% agarose gel. The qPCR was performed with an iCycler IQ5 (Applied Biosystem).

Kolb, S., Knief, C., Stubner, S., Conrad, R., 2003. Quantitative detection of methanotrophs in soil by novel pmoA- targeted real-time PCR assays. Appl. Environ. Microbiol. 69, 2423–2429. https://doi.org/10.1128/AEM.69.5.2423


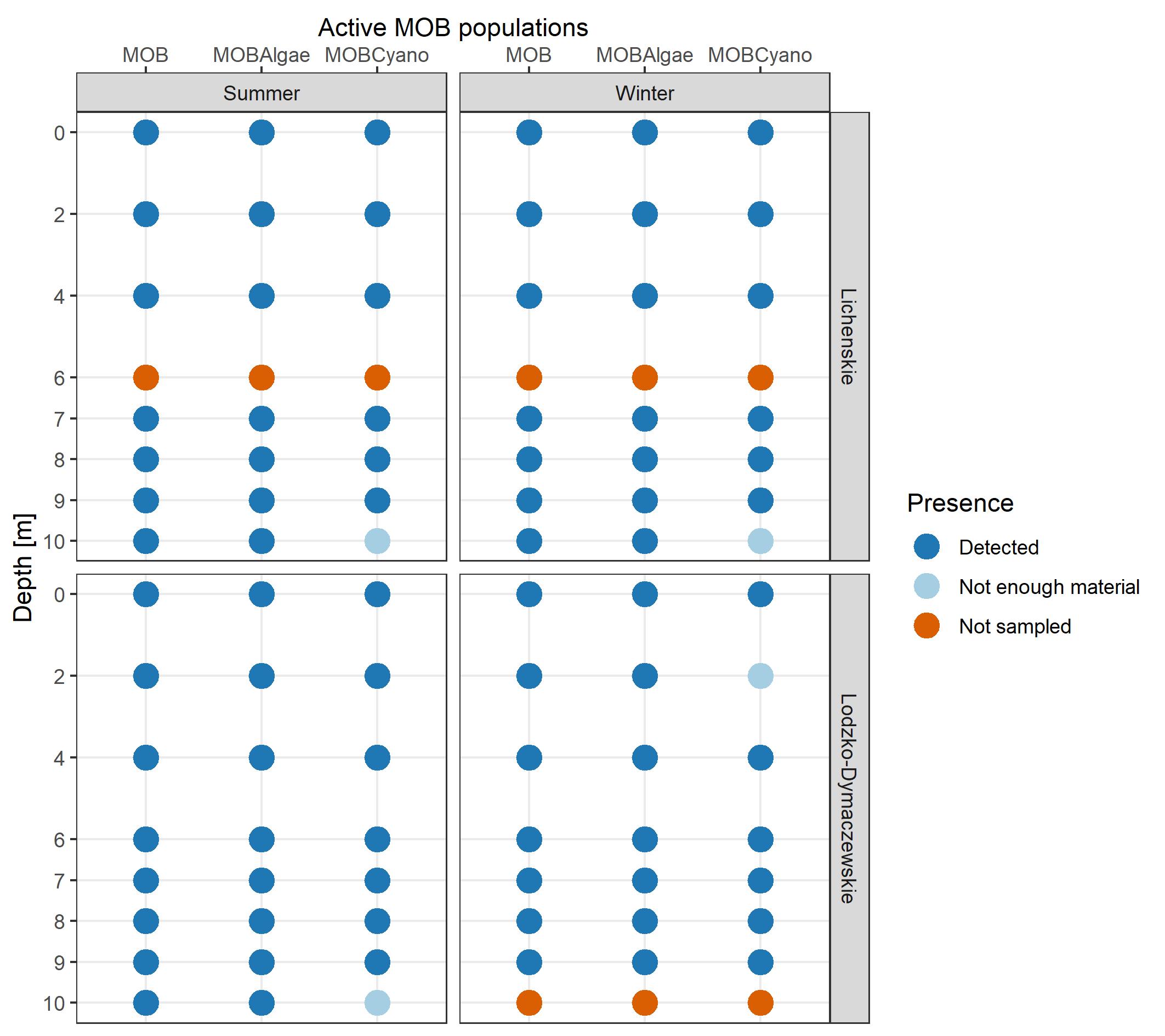


**Fig. S.3** Plot depicting the number of sorted events related to the 3 active MOB populations of interest: MOB (free-living), MOBAlgae and MOBCyano in Licheńskie (upper side of the figure) and Łódzko-Dymaczewskie (lower side of the figure) Lakes. Samples collected in summer are shown on the left side of the figure and samples collected in winter are shown on the right side of the figure. Not enough material means that the sample was processed, but it was not enough to sort all the fractions*.*


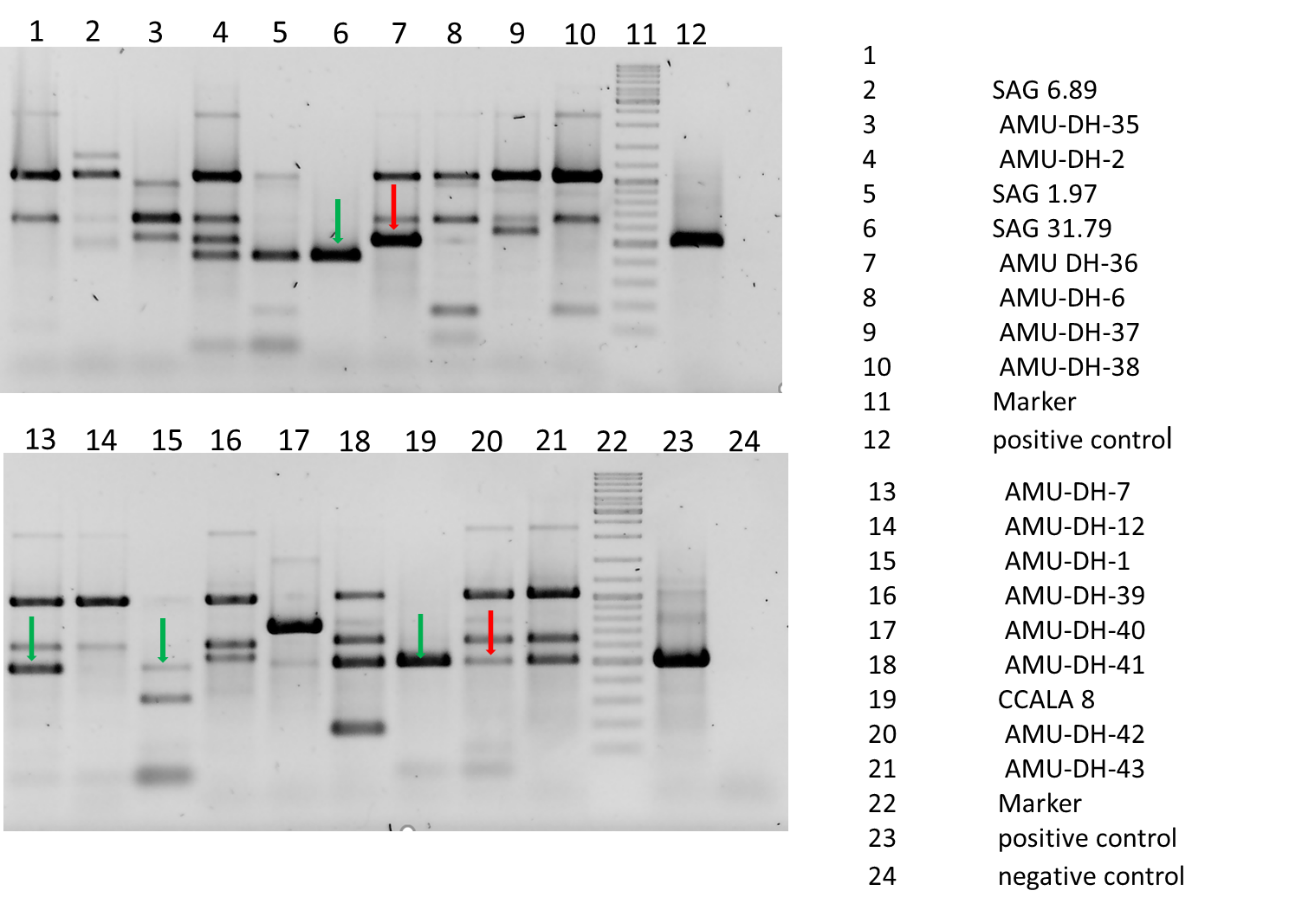


**Fig. S.4** Gel electrophoresis of PCR products of *pmoA* gene detection in genomic DNA extracted from cyanobacterial cultures. All bands of the size similar to positive control were cut out for further analyses, but only 6 of them (labelled with arrows) gave enough DNA for sequencing. Green arrows mark bands that gave positive results, and red ones failed.
